# New Products

**DOI:** 10.1155/S1463924684000110

**Published:** 1984


Journal of Automatic Chemistry, Volume 6, Number 1 (January–March 1984), pages 47–60

### New Products

### Automated peak height analysis

Compatible with almost any analyser which produces a regular series of peaks, DMA's PHA-82 could save a significant amount of time in laboratories where a large throughput of samples needs to be analysed for peak height, slope, and other parameters. Results can be stored on disk and, if required, printed onto laboratory note forms.

Based on the Commodore CBM/PET computers the PHA-82 can handle up to 16 channels of analogue data; a machine code operating system manages an A/D interface and combines all the necessary logic for measuring and displaying the peak data in real-time. A double computer package is available—one computer continuously analyses the analogue signals, and the other is free to calculate the results, edit the data and enter details of the next batch.

*Full information from Digital Measurement & Analysis, 906 Woodborough Road, Nottingham NG3 5QR, UK. Tel.: 0602 622686.*

Circle No. 1 on Reader Enquiry Card

### Laboratory working conditions

A unique, combination special stains and cover-slipping bench using Corian worktops and under-bench extraction has recently been installed in the histology laboratory of the North Manchester General Hospital. The benchtop, completely fabricated from the tough, nonporous man-made stone developed by Du Pont, represents a major design advance.

The continuous laboratory top incorporates two shallow Corian sinks for slide washing and staining; two removeable trays for staining pots with individually controlled extraction beneath; and two flush, perforated section/cover-clipping panels, also removeable with extraction beneath. Corian is a solid product, which cannot delaminate, and is simply glued together to provide a seamless surface with completely impermeable joins. Potential contamination traps have been virtually eliminated and all areas can be kept clinically clean. Staining agents are readily removed with ordinary cleaning materials. It is available in sheet form in 6 mm thickness for vertical applications or 13 mm and 19 mm thickness for horizontal use. It also comes in a variety of moulded sinks and basins which can be bonded to become integral to an overall worktop.

*More information (UK) from C. D., Richmond House, 16 Blenheim Terrace, Leeds LS2 9HN, UK. Tel.: 0532 439651; (other territories) Du Pont de Nemours International S.A., PO Box, CH 1211 Geneva 24, Switzerland.*

Circle No. 2 on Reader Enquiry Card

### Process control

At the Interkama exhibition in FR Germany, which was held in mid November 1983, Fisher Controls demonstrated a complete process control capability for the process and energy industries.

PRōVOX batch instrumentation was shown for the first time. It features the co-ordination of the batch cycle, integrated discrete and regulatory control, complete recipe handling, easy operator interface and complete process management. Up to 20 recipes can be stored in the PRōVOX batch console and a further 125 on disk; recipes are downloaded to controllers, allowing rapid change of feeds, grades and even types of product. PRōVOX batch is intended for application in the chemical and pharmaceutical industries and in food and beverage processing.

Another new product was the DV series Vortex flow transmitter—a rugged instrument that measures volumetric flow of liquids, gases and superheated steam. Fisher Controls claim that it provides the accuracy of a turbine or positive displacement flowmeter, yet offers the many fluid applications, simplicity of calibration and economical advantages, of an orifice plate flowmeter.

Also shown for the first time in Europe was the Type 2390 liquid level transmitter which is mounted on the established 249 series LEVEL-TROL displacement sensor. The transmitter features ease of calibration in that the zero, span and dry span calibration adjustments can all be done externally, even in hazardous areas. The use of Hall-effect sensing gives clean, noise-free signals and enables the measurement of small specific gravity changes, permitting its use in density and other applications requiring low levels of input signal change.

*Reader enquiries to M. W. Mason, Sales Director, Fisher Controls Ltd, B1 Century Works, Conington Road, Lewisham, London SE13 7LN.*

Circle No. 3 on Reader Enquiry Card

### Non-destructive analyser for metallic coatings

A broad spectrum system for analysing metallic coatings has been developed by Philips for the general coatings industry. COAT 95 is based on X-ray excited emissions and can cope with coatings of thicknesses ranging from a few angstroms up to multielement or multilayer finishes extending well into the micron range. Measurements can be made on silicon wafers, lead frames or any other device—from the largest-diameter wafer down to one millimetre-square samples. Multielement coatings may be of any combination of elements and a complete analysis can be made within a few minutes. The process is completely non-destructive and the results highly accurate.

COAT 95 is simple to operate, with single-button operation for on-line daily use. It is a software system for use with the Philips PV 9500 automated energy-dispersive spectrometers, which employ DEC LSI-11 computers. The instruments produce full information on both coating composition and thickness in the form of fast-colour spectral displays.

*Details from Pye Unicam Ltd, York Street, Cambridge CB1 2PX, UK. Tel.: 0223 358866.*

Circle No. 4 on Reader Enquiry Card

### Zorbax and HPLC

A rapid analysis, small particle (3 $\mu$m) column for high performance liquid chromatography was introduced by Du Pont in mid October 1983. The Zorbax Golden series column is able to separate samples two to three times faster than conventional analytical columns and improves resolution. There are three types of column: ODS (C18), C8 and SIL (silica).

The high performance of Golden Series columns apparently results from the inherent properties of Du Pont's 'Zorbax' particle, which is specially designed for high performance liquid chromatography. While the efficiency of a column rapidly increases as the diameter of the packing particle decreases, the problem with 3 $\mu$m particle has been poor particle size control leading to a broad size distribution. These liabilities result in excessive backpressures and decreased column lifetimes. With Zorbax technology, Du Pont have created a spherical, mechanically strong particle with narrow-size distribution—the company maintain that these properties under a unique, tightly controlled manufacturing process.

The columns are shorter (8 cm) but wider (6·2 mm i.d.) than a standard column of the same sample capacity: this internal volume allows use of standard injectors and detectors, and no sensitivity is lost due to either decreased sample size or detector cell path length. So Golden Series columns do not require highly specialized LC components.

The new column also operates effectively at both elevated and ambient temperatures.

*Further information from Du Pont de Nemours International S.A., PO Box, CH 1211 Geneva 24, Switzerland.*

**Circle No. 5 on Reader Enquiry Card**

### HPLC autosampler

The model 504, Beckman's new autosampler, can hold up to 102 samples and was designed so that the column is mounted very close to the point of sample injection. This optimizes the performance with high-speed columns by minimizing peak broadening. The 504 can inject 1 $\mu$l from a total sample volume of 10 $\mu$l; sample volume is adjustable from 1 to 100 $\mu$l and the autosampler can store up to nine separate programs.

A time-saving feature is that the 504 allows access to samples at all times so the first few samples can be loaded, the system started and these samples analysed while loading is continued. A unique washing feature allows trace analysis work to be carried out with minimum cross contamination. The model 504 can be used in conjunction with Beckman liquid chromatographs and with those of other manfuacturers.

*Details from Beckman-RIIC Ltd, Progress Road, Sands Industrial Estate, High Wycombe, Buckinghamshire, UK. Tel.: 0494 41181.*

**Circle No. 6 on Reader Enquiry Card**

### Linear actuator

Linak is a low-cost linear actuator for 12 V and 24 V d.c. operation, which was designed for linear movement requirements of both indoor and outdoor installations.

The stainless-steel piston, and the alkyd varnish treatment of the surfaces of all other parts ensure that the actuator remains free from dirt and water. The outer surfaces are weather-proof. For open outdoor installation the actuator is provided with a rubber mantle for additional protection. It is maintenance free and protected against mechanical overload. It can be controlled by either a double-pole rocker switch or an electronic end-stop switch. Both of these types of control unit eliminate the need for end-stop: this results in definite savings, in addition to the fact that only two electric leads are required for the actuator.

A built-in potentiometer permits automatic positioning. The potentiometer is synchronized to the movements of the piston, which ensures that the piston's movement is under control throughout the entire stroke. The potentiometer and the servo control system mean that positioning accuracy of up to $\pm$0·1 mm is attainable. The range of Linak linear actuators provide a thrust of up to 3000 N and speeds of up to 39 mm/s. Standard strokes range from 50 to 400 mm.

*The manufacturer is Unimatic Engineers Ltd, Granville Road Works, 122 Granville Road, London NW2. Tel.: 01 455 0012.*

**Circle No. 7 on Reader Enquiry Card**

### Ultrasound in chemistry

Polysonics's (USA) Doppler ultrasonic flowmeter systems are to be sold in the UK by Gendeal Ltd. The systems are capable of measuring flow rates in full pipes from 1″ upwards; they are non-invasive and can be fitted quickly to all materials bar rubber and concrete. They are suitable for measuring the flow of slurries, waste effluents, cooling water, spent acids, caustics and so on and the transducers, which strap onto the outside of the pipe, will withstand temperatures in the range −300°F up to +320°F. There are eight models in the range including dedicated, explosion proof and portable types. Most provide a 4-20 mA isolated output for chart recorders or other instrumentation and optional alternative outputs are also available.

The standard read-outs are in terms of velocity in ft or m/s and knowing the internal diameter of the pipe, the flow in volume per unit time can be calculated. Dedicated units can be graduated in engineering units of flow if required; totalizers and signal strength meters can also be fitted.

Of the two portable units, the UFM-P and UFM-PD, the former is housed in a robust casing suitable for use in harsh environments and for long periods; the UFM-PD is housed in a suitcase-type enclosure and is designed for diagnostic use on a short-term basis. This instrument is equipped with a flow-rate indicator, totalizer, signal strength meter and a built-in calculator for speed and ease of use.

Whereas most of the units employ one transducer, for use with cleaner liquids, Models DHT and DHT-P are offered—these use a dual head transducer for effective and accurate measurement.

*Full details from Gendeal Ltd, 8 Kingsbridge Road, Walton-on-Thames, Surrey KT12 2TL, UK.*

**Circle No. 8 on Reader Enquiry Card**

### Computer balance

The Fisher/Ainsworth MX-400 electronic balance has four built-in weighing modes (0·01 to 400 g, 0·001 to 14 avoirdupois ounces, 0·1 to 6173 grains and 0·0001 to 0·88 avdp. pounds), resolution to 0·01 g and a 3 s response. An optional interface box and cable permit connection to any RS232C-compatible computer or printer for automatic print-out. The balance can be commanded to tare, weigh or change functions by remote computer control; also to count parts, determine weight gain-or-loss percentages, and give a read-out in any user-defined units.

*Fisher describe the balance in their Bulletin No. 674—copies from Fisher Scientific Company, 711 Forbes Avenue, Pittsburgh, Pennsylvania 15219, USA (in Canada contact Fisher at 112 ch. Colonnade Road, Neapean, Ontario K2E 7L6).*

**Circle No. 9 on Reader Enquiry Card**

### Dual-range balance

Bulletin No. 639 describes another new Fisher/Ainsworth product: model LC-5500. It offers dual-range capability with weighing to one part in 50 000 in either range—it can be a 'workhorse' 5000 g balance with 0·1 g resolution or a sensitive 500 g balance with read-out to 0·01 g Copies of the Bulletin from Fisher (above).

**Circle No. 10 on Reader Enquiry Card**

### Stirrer

The T & M electronic stirrer has a speed range of 200 to 1000 rpm, it is motorless and cannot stall at any speed. Speed control is provided by voltage-regulated circuitry and a speed indicator; it is recommended for repetitive tests. Automatic acceleration provides complete control of the stirrer bar when changing speed and after switching on, when set at high speed.

The standard bench model provides the necessary circuitry to power two remote units; outlet sockets are required for the remote units which are fitted to order. A range of remote units is offered : from a small low-power unit for use in confined spaces, up to a version which gives the same power output as the master unit.

*More information from Townson & Mercer Ltd, Chadwick Road, Astmoor, Runcorn, Cheshire WA7 1PR, UK. Tel.: 09285 76245.*

Circle No. 11 on Reader Enquiry Card

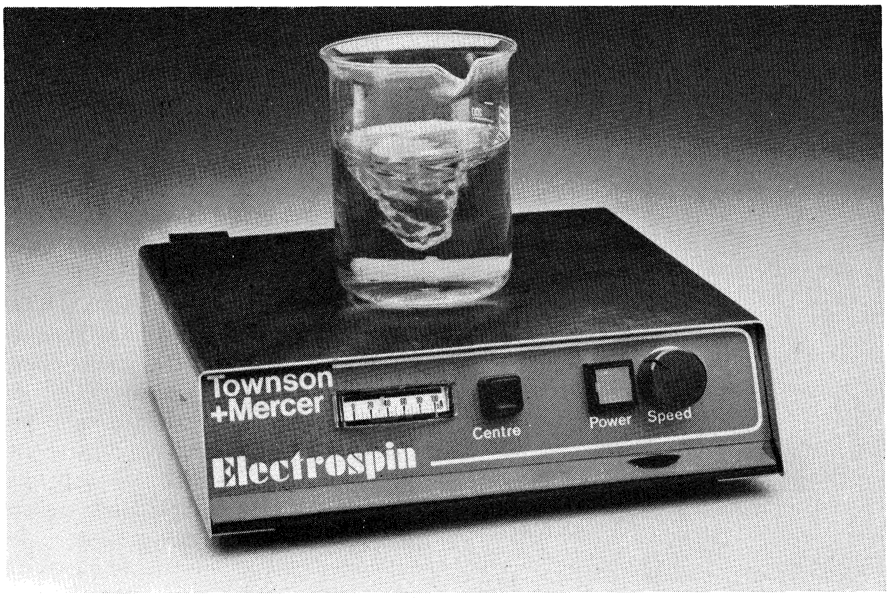

*Electronic stirrer. Dimensions are 215 mm × 215 mm × 55 mm deep, weight is 1·5 kg. A wide speed range permits stirring of both large and small volumes and high and low viscosities.*

### Dynamic gas blending

The multichannel Dyna-Blender is the third generation of Matheson's gas-blending equipment. It delivers on-line, premixed gases at different ratios regardless of temperature, pressure or flow variations. The unit features up to four channel blending capability, a digital display and separate controls for each channel. It also offers costs savings and space savings over modular systems without compromising control, accuracy and precision.

Each of the four channels can be controlled, remain independent with a constant flow, or remain idle with no flow. Control of the channel can be by an external ratio of another flowstream, by internal manual adjustment, or by an external 0–5 VDC signal. The digital LED display and the four channel set-read switch allow the operator to monitor each channel, one at a time, as desired.

*Further information from Matheson Gas Products, Inc., 30 Seaview Drive, Secaucus, New Jersey 07094, USA.*

Circle No. 12 on Reader Enquiry Card

### Process oxygen analyser

The new 1100A analyser has two parts: a transducer unit and a control unit; these can be mounted up to 300 m apart, the functions of the transducer unit being controlled and monitored through the control unit's keyboard.

Both units were designed to be used in Zone II/Division 2 hazardous areas, and a version of the transducer unit is suitable for Zone I/Division 1. Gas samples can be hazardous, oxygen enriched; certification has been applied for.

Supplied as standard is an isolated 4-20 mA output with the microprocessor giving a choice of seven spans and a zero-suppression facility to 99%, providing several hundred output ranges. The control unit includes a digital LCD display, reading from 0–100% oxygen to 0·01% resolution, and also supplies the power requirement for the transducer unit. The memory is protected by battery back-up.

Options available include alarm relays for the built-in oxygen level alarms, self-diagnostics, sample flow alarm and pressure compensation. An RS232C or current loop digital interface can be specified, which allows the 1100A to be controlled by a computer. Sample-handling systems to deal with wet or dry gas can be supplied and special measuring cells to resist most organic solvents or corrosive vapours are available.

*Details from Servomex Ltd, Crowborough, East Sussex TN6 3DU, UK. Tel.: 08926 2181.*

Circle No. 13 on Reader Enquiry Card

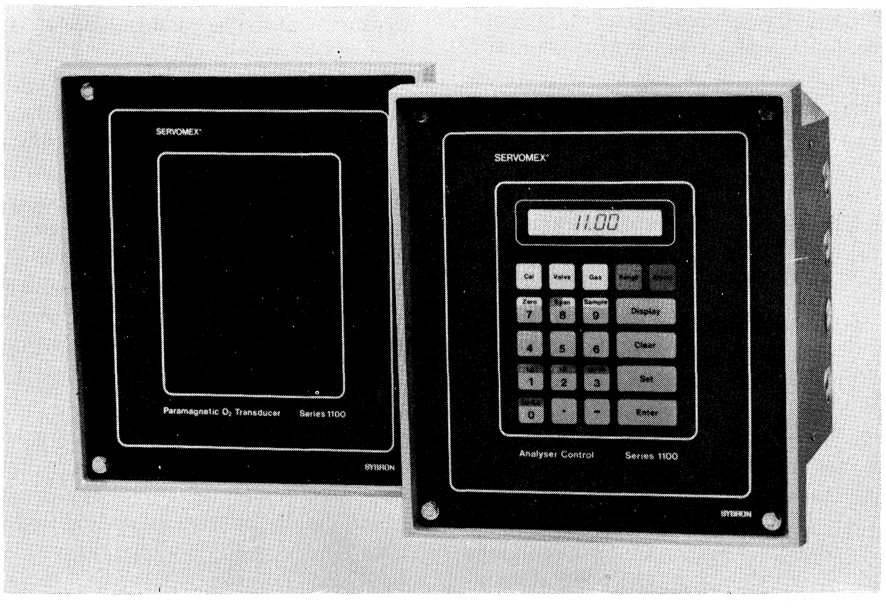

*The 1100A Industrial Process Oxygen Analyser. Options include auto-calibration or auto-check, which can be set through the keyboard for initiation at predetermined intervals or activated as required. (Servomex Ltd, Crowborough, UK.)*

### Perkin-Elmer 8300

A compact, easy-to-use and moderately priced gas chromatograph has been announced by Perkin-Elmer. The 8300 is intended for routine or process laboratories.

The chromatograph has automated bleed compensation; optional real-time display of a chromatogram; and optional integral data-handling facilities. The system is controlled by an advanced microprocessor, with user-interaction through a 12 in visual display unit and simple keyboard with eight 'soft keys'.

The screen graphics and data handling options allow the 8300 to be developed into a complete chromatography and data-processing system. Chromatograms can be displayed in real time on the screen and replotted at the end of the run using variable screen widths and attenuations. Hard-copy print-outs can be obtained by connecting the machine to any pen recorder or to a printer/plotter.

At the end of an analysis, the data-handling system can display results in the form of a peak table and allow post-run manipulation of the data. Via the graphics printer, the user can recall the chromatogram to print in peak names and retention times on selected peaks. Inexperienced users can be guided through the data-handling system by prompts; a 'short-cut' mode of operation is also possible.

Automated bleed compensation (ABC) has many advantages over the more generally used dual-column balancing techniques for temperature-programmed FID operation. It minimizes setting-up time and reduces costs. Once the column has been conditioned, bleed correction is obtained by a simple calibration procedure. After set-up, it is even possible to modify the temperature programme without re-calibration. Furthermore, ABC can be used to temperature program packed columns with specific detectors, such as the electron capture, or to program capillary columns at near maximum detector sensitivity with negligible base-line shift.

The chromatograph is available in two series: the 8310 for packed column operation; and the 8320 for dedicated capillary systems.

The 8300's oven is optimized for capillary column use, but is equally suited for packed columns. Unusual accessibility is achieved by a unique design of analyser which slides forward, pulling the columns out of the oven to give all-round access to the column connections. The oven is compact, but holds 2 × 4 m eighth inch o.d. metal or glass-lined stainless-steel columns, 2 × 2 m quarter inch o.d. columns, or a 1 × 4 m system. Isothermal, single or dual ramp temperature-programmed operation are possible; with the addition of a sub-ambient accessory, the instrument can be operated down to 0°C to improve separations of mixtures containing volatile components.

A range of easily interchangeable detector and injector modules is available. There are six detector options for the 8310: single FID, ECD, NPD, HWD, dual FID and NPD/FID; the 8320 series has single FID, ECD, NPD and FID/NPD options are available. A choice of pneumatic control panels for the 8310 provides cost-effective control for carrier gas and detector gas supplies, while, for the 8320, the specially designed pneumatics with electronic pressure read-out on the screen allow precise setting of carrier gas supply, even at very low flow rates. The range of injectors for the 8310 series includes single or dual packed for $\frac{1}{8}$ in or $\frac{1}{4}$ in columns and a split/splitless capillary injector; the 8320 series of dedicated capillary chromatographs are available with split/splitless and on-column injectors.

*For further information contact Perkin-Elmer Ltd, Post Office Lane, Beaconsfield, Buckinghamshire HP9 1QA, UK. Tel.: 04946 6161.*

Circle No. 14 on Reader Enquiry Card

### Electrode system

A withdrawable steam-sterilizable electrode system for pH measurement of industrial fermentation processes has been introduced by Kent Industrial Measurements. The stainless-steel EIL Model 2890 is designed for use in sealed vessels at pressures up to 11·25 kgf/cm$^2$ and at temperatures up to 150°C, or vacuum vessels with a maximum operating vacuum of 0·105 kgf/cm$^2$. It features a withdrawable valve-mounting assembly, which enables the electrode to be removed from the reaction vessel or pipe, and changed and sterilized in its own housing without interrupting the process. The holder is withdrawn by an operating screw on the outside of the assembly. In the withdrawn position the electrode is completely sealed in the housing and a jet of steam is introduced via a port in the base of the holder.

The holder consists of an electrode stem holder with optional electrode guard, and an upper chamber featuring a 'sight' glass arrangement for checking the electrolytic level of the electrode. Connections for an air supply and pressure gauge allow pressurization of the upper chamber to 2 psi above the sample pressure at the electrode tip.

The electrode used is Kent's 1170 combination pH glass electrode. It is supplied filled with glycerol/potassium chloride electrolyte, together with spare 'top-up' solution. A glycerol-based solution is used to stop the reference solution boiling during sterilization.

To minimize errors in pH measurement due to temperature variations in the reaction vessel, a separately mounted temperature compensator is available.

*Details from Kent Industrial Measurements Ltd, Analytical Instruments, Hanworth Lane, Chertsey, Surrey KT16 9LF, UK. Tel.: 09328 62671.*

Circle No. 15 on Reader Enquiry Card

### CAMAG Reprostar

There is now a transilluminator to go with the CAMAG Reprostar: a machine that photographs chromatograms, electropherograms etc. in visible light, and under long-wave and short-wave UV light. The transilluminator means that Reprostar users can assess and photograph, in 300 nm UV light, transparent objects such as RNA/DNA fragments stained with ethydium bromide after gel electrophoresis. (The transilluminator can be retrofitted to all Reprostars.)

*For literature contact CAMAG, Sonnenmattstrasse 11, CH-4132 Muttenz, Switzerland. Tel.: 061 613434.*

Circle No. 16 on Reader Enquiry Card

### Chromatography Discussion Group

In 1982 the Chromatography Discussion Group celibrated its Silver Jubilee, having spent the previous 25 years promoting the interests of the international chromatographic community. The group now has the resources to embark on a campaign to encourage young chromatographers to become members: chromatographers under 25 will be able to join for a reduced subscription of £6·00 per annum (1984). It is hoped that this heavily subsidized fee (normal membership is £18·50) will attract a number of young chromatographers to enjoy the benefits of the Group and, hopefully, advance their careers.

*More information about this scheme and about the Group's Meetings, Abstracts and Symposia from the Chromatography Discussion Group, Trent Polytechnic, Burton Street, Nottingham NG1 4BU, UK.*

Circle No. 17 on Reader Enquiry Card

### Microprocessor-based automatic sample injector for liquid chromatography

Designed for unattended repetitive autosampling, as well as fast single sample injections, the LDC/Milton Roy Model 713 was launched at the end of August. The 713 can inject 60 samples with up to three injections per vial, giving a total of 180 possible injections. It greatly reduces the amount of sample required for injection: just 100 $\mu$l is required for a 10 $\mu$l injection. The 713 is simple to operate, with a data-entry system featuring an LED visual display coupled to a keyboard. Programmable functions include cycle time, last bottle to be sampled, flush time, injections per vial and timed external commands. The sample injector is offered with a choice of injection valves: the Rheodyne 7010A, 7126 and 7410A (sample loops of 0·5 $\mu$l to 200 $\mu$l are available).

*Details from Laboratory Data Control (UK) Ltd, Milton Roy House, High Street, Staffordshire ST15 8AR, UK. Tel.: 0785 813542.*

Circle No. 18 on Reader Enquiry Card

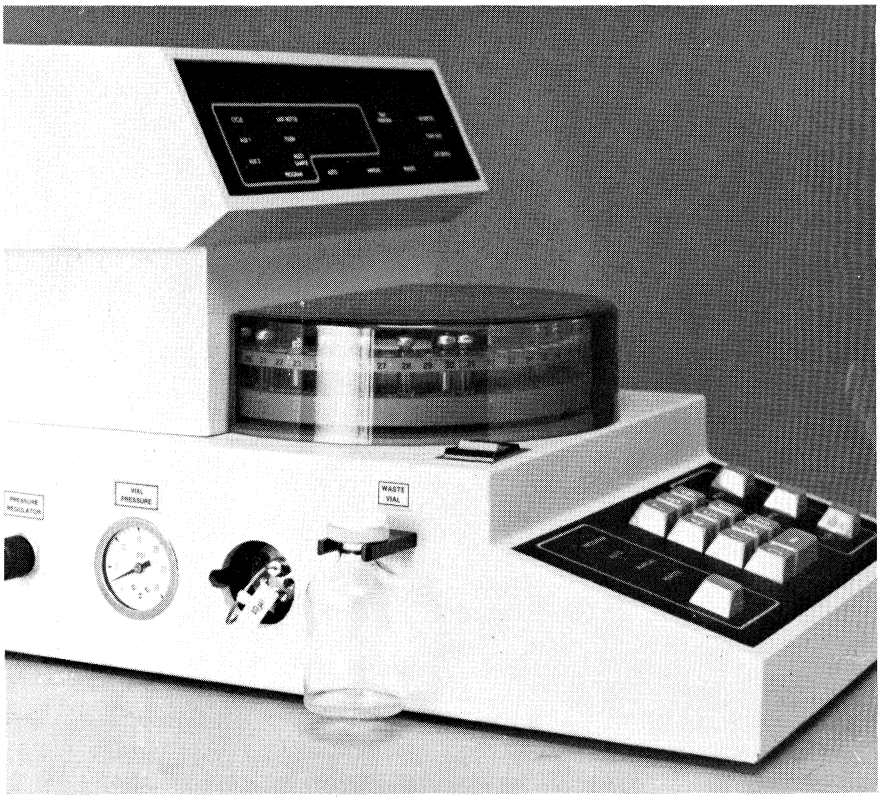

*The Model 713 from Laboratory Data Control (UK) Ltd. The sample injector can be controlled remotely by laboratory controllers or computers and will perform with virtually any LC system.*

### Perkin-Elmer GC

SIGMA 300 has been added by Perkin-Elmer to their range of gas chromatographs. Based on the SIGMA 3B, it is a compact, multi-detector, single- or dual-column instrument, capable of handling a variety of samples.

There are seven detector options: ECD, FID, HWD, NPD, FPD, PID and the Hall electrolytic conductivity detector; up to three detectors may be installed and operated simultaneously. There is a choice of packed column, split/splitless capillary column and on-column capillary injectors. Both packed and capillary injectors may operate simultaneously. Automatic control of a gas sampling valve of the split/splitless capillary injector is standard. Packed injectors are supplied with both $\frac{1}{8}$ in and $\frac{1}{4}$ in fittings.

The SIGMA 300 offers two versions of flow control for packed column carrier gas: a new, low-cost flow control, or digital flow control. There is also a choice of manual or automatic capillary pneumatics.

Optional RS232C-compatible computer communications allow parameters such as oven, detector and injector temperature to be set and monitored from an intelligent terminal or a large computer system. This enables a network of SIGMA 300 units to be set up, providing a cost-effective means of implementing laboratory automation.

The 300 can be custom-built to meet specific needs. Alternatively, preconfigured units, based on the most popular configurations, are available: a dual FID, and FID/HWD, an ECD and an HWD for packed column operation, plus an FID for capillary operation. Any of these dedicated units can be modified at a later date with different detectors and injectors.

The versatility of the gas chromatograph is further extended by the range of accessories available. These include the HS-6 automatic headapace sampler and the Model ATD 50 automatic thermal desorption system. The new AS-300 autosampler, which can be installed directly onto the SIGMA 300, increases sample throughput and forms part of a cost-effective, automated GC system for routine applications.

*For further information contact Perkin-Elmer Ltd, Post Office Lane, Beaconsfield, Buckinghamshire HP9 1QA, UK. Tel.: 04946 6161.*

### *FTIR application booklet*

An application booklet on the Perkin-Elmer 1500 Series of Fourier Transform infra-red spectrophotometers, the Models 1500 and 1550, is now available. After a brief description of the 1500 Series, the booklet covers experiments that demonstrate the versatility of the instruments in a wide range of applications areas—from routine analysis to the most demanding analytical requirements of the modern laboratory. Copies are free from Perkin-Elmer (above).

Circle No. 19 on Reader Enquiry Card

### Field or lab pH meter/thermometer

A high specification, digital pH meter and thermometer has been announced by Kane-May. The KM 7002 offers automatic and a good pH accuracy to plus or minus 0·02 pH, as well as a resolution of 0·01 pH using most combination electrodes. It can measure temperature from $-30$°C to $+450$°C to an accuracy of plus or minus 0·7% of reading. Resolution is 1·0°C. The KM 7002 can operate with any of Kane-May's standard range of 40 temperature probes.

To allow for non-pH electrodes, or other millivolt inputs, the KM 7002 can also display MV from $-1999$ to $+1999$.

*More information from Kane-May Ltd, Burrowfield, Welwyn Garden City, Hertfordshire AL7 4TU, UK. Tel.: 07073 31051.*

Circle No. 20 on Reader Enquiry Card

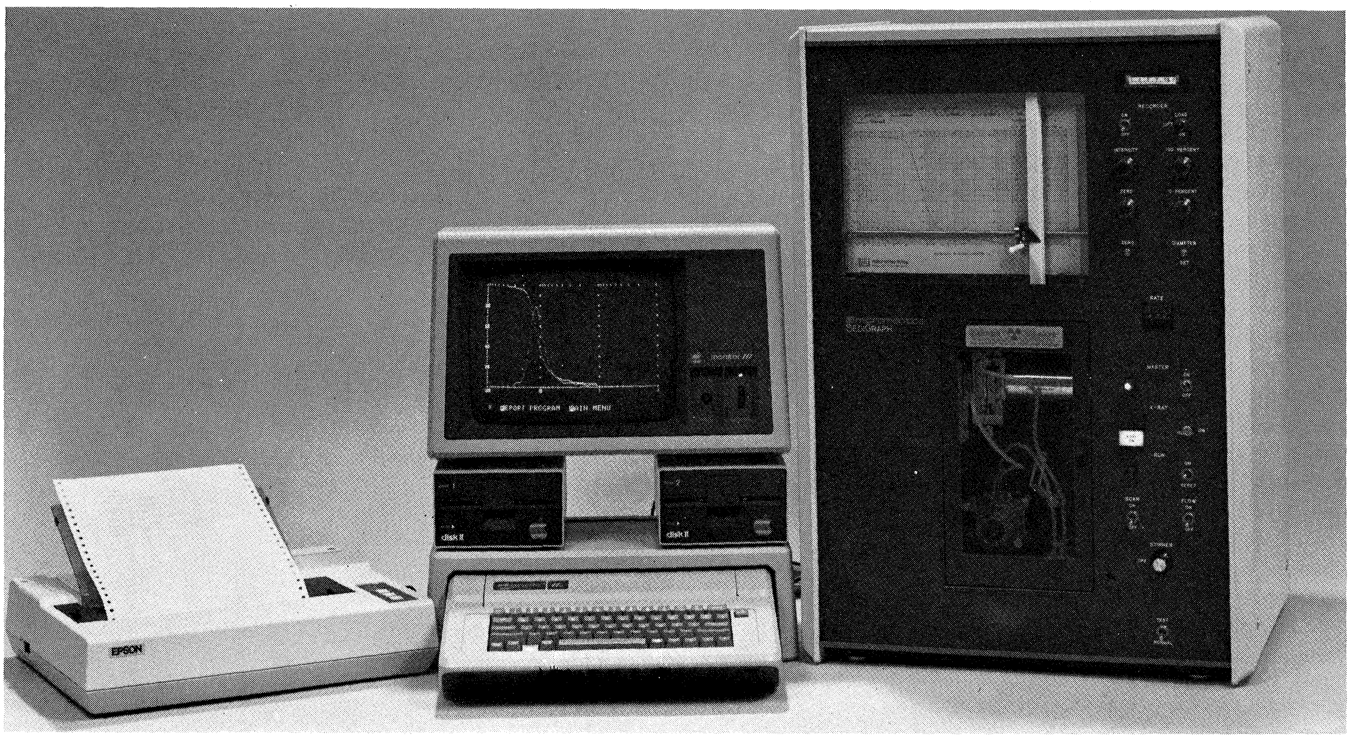

*Sedicomp—a computer-to-Sedi-Graph interface designed to allow any Sedi-Graph 5000 ET to be adapted to Apple II micros. The interface expands the SediGraph data output capability so that it can provide tabular and graphic data in printed or video display format. Sample identification and analysis calculations are apparently greatly improved using the computer system. Programs and analysis data are stored on standard 5-¼ in floppy disks for fast data recall and comparison of analyses.*

*The SediGraph automates the classical sedimentation technique and is noted for its wide analysis range. It can analyse particle sizes in the range from 0·1 μm to 100 μm in a single analysis that accounts for the entire sample, including diameters outside its measurement range.*

*The SediComp package includes Apple PC boards, master program disks, connecting cables and a user manual. Details from Micromeritics Instrument Corporation, 5680 Goshen Springs Road, Norcross, Georgia 30093, USA. Tel.: 404 448 8282.*

Circle No. 21 on Reader Enquiry Card

### DAPS

A data acquisition and processing system has been developed by Stanton Redcroft Ltd for handling thermally derived data. DAPS 2 can monitor, store and display data from four sensors with a resolution 20 times greater than conventional chart recorders; it can also plot expanded portions, or the entire results, as graphic colour curves on A4-sized paper.

The data collected can be manipulated on a video screen to obtain a detailed understanding of thermal events. In addition, analysis programs included in DAPS 2 offer automated calculation of a wide range of thermal parameters, from results obtained with thermobalances (TG), differential thermal analysers (DTA), differential scanning calorimeters (DSC), thermomechanical analysers (TMA) and instruments that combine the functions of a TG and DTA, or a TG and DSC.

The system is based on a Commodore PET 4032, which collects test variables via a four-channel IEEE 488 Data Acquisition Unit with a resolution of 1 : 20 000. This high resolution reduces the need for preliminary experiments, for example to determine approximate weight losses or the dynamic range of DTA peaks. Raw data are simultaneously stored on a high-speed digital tape recorder (which acts as a non-volatile extension to the computer memory) and displayed continuously on the VDU. At the end of each test run data is transferred to the disk system, where it is permanently stored for future reference. Whilst the selected plot is displayed, DAPS 2 offers two further choices of expanding regions of interest by use of a movable cursor on the VDU and printing the expanded region or the entire plot on a Houston Instruments' HIPLOT EDMP-4M443 A4 six-colour graphics plotter. Hardware is compatible with Stanton Redcroft's STA 780, TG 760, TMA 790 and DTA 670 series of instruments.

The DAPS 2 allows the collected data to be manipulated in a variety of ways, for example in the determination of peak temperature, extrapolated onset and offset times and temperatures, weight loss percentage, peak area integration, peak shoulder identification and TG buoyancy correction routines.

In addition, the software package can be used for more specialized applications, such as oxidative inductive stability, percentage crystallinity, degree of cure, glass transition temperatures and identification of maximum temperatures in highly exothermic reactions.

A pack of 10 floppy disks, supplied with the system, offers sufficient memory storage for records of approximately 400 tests to be kept on file. For installations where a stable mains supply is not available, a voltage-stabilizer/interference suppressor can be provided as an optional extra.

*Enquiries to Stanton Redcroft Ltd, Copper Mill Lane, London SW17 0BN. Tel.: 01 946 7731.*

Circle No. 22 on Reader Enquiry Card

### Chromatography software

GCDS is a chromatography analysis system, which offers a range of capabilities to give analysts automated measurement of a chromatogram. Providing unlimited storage of methods and chromatogram raw data, and the flexibility of post-acquisition processing, GCDS is described as out-performing any computing integrator. Designed to run on Apple II microcomputers, the system was written to be easy to use, requiring only a single key press for many entries.

After the initial creation of a method and the subsequent acquisition of the chromatogram raw data in real time, the method can be tailored to suit the acquired chromatogram, without the need to rerun a sample. The tailored version of the method can be stored and used for later acquisitions.

*More information from U-Sci Ltd (they are a subsidiary of U-Microcomputers and produce a range of products for the Apple) at Winstanley Industrial Estate, Long Lane, Warrington, Cheshire WA2 8PR, UK. Tel.: 0925 54117.*

Circle No. 23 on Reader Enquiry Card

### HPLC data system

At Laboratory 83 (October, London) Beckman launched their model 450—their latest data system/controller, which is intended for use in HPLC as a methods development and automation centre for a wide range of research and industrial applications (for example pharmaceuticals, food, chemicals, oil and plastics). The system provides up to four channels of HPLC control, independent multichannel data acquisition, data reduction, storage and management. It is expected to be of special interest to laboratories using more than one liquid chromatograph, and is fully compatible with existing Beckman/Altex modules and systems.

Special software packages include an option for storage and manipulation of spectral data from the Beckman Model 165 Detector providing more positive peak identification, a user BASIC package, and GPC calculations.

The operating system is disk based, so new software updates can be undertaken easily at little additional expense.

The 450 has been designed for simple use by laboratory staff and provides more information on each analysis than is obtainable by manual methods. Up to four chromatograms can be viewed on the CRT simultaneously and raw data and results can be stored on disk with production of a hard copy print-out when required.

As an HPLC control centre, the system eliminates the need for individual pump controllers and integral automation allows unattended operation of other HPLC modules—autosamplers and switching valves for example. It has 160 k bytes of RAM memory expandable to 288 k bytes and features dual floppy disks providing 640 k bytes of usable storage for methods and data. Accessories include data channels, instrument control channels, a bi-directional RS232C port and a pneumatics interface.

*Details from Beckman-RIIC Ltd, Progress Road, Sands Industrial Estate, High Wycombe, Buckinghamshire, UK, Tel.: 0494 41181.*

Circle No. 24 on Reader Enquiry Card

### Gas chromatograph

When Analytical Instruments Ltd was set up in 1967 the basis of the business was to exploit detectors developed for gas chromatography and to use them in special-purpose instrumentation. These products have incorporated the thermal conductivity detector for leak detection and atmosphere monitoring, the electron capture detector for leak detection, atmosphere monitoring and specialized vapour detection systems and thermionic ion emission for refrigeration gas detection. The company is probably the largest manufacturer in the world of electron capture detectors—over 10 000 have been made.

AI entered the gas chromatography market three years ago with the AI 90 Gas Chromatograph and has now introduced the AI Model 92.

The AI 92 is announced as having outstanding thermal performance in terms of temperature control, minimal column oven gradients, rapid cooling and reproducibility of temperature program profiles. Much attention has apparently been given to design—user control, for example is via a keyboard allowing immediate commands to be executed in the same way as a knobs-and-dials instrument, but with the benefit of preprogrammed analysis routines.

*For further details contact Analytical Instruments Ltd, London Road, Pampisford, Cambridge CB2 4EF, UK. Tel.: 0223 834420.*

Circle No. 25 on Reader Enquiry Card

### GAMBICA

The association for the instrumentation, control and automation industry in the UK, GAMBICA, is reviewing its criteria for membership in order that non-British manufacturers will be able to join. The association's president, Bob Eade, Chief Executive of Thorn EMI Technology, believes that the change is important because the business is becoming increasingly international and 'all companies having an important place in the UK economy can be involved with the collective voice of the industry'.

*Information about this and about the association's other plans from Desmond Cavanagh, GAMBICA, 8 Leicester Street, London WC2H 7BN. Tel.: 01 437 0678.*

Circle No. 26 on Reader Enquiry Card

### 'Flow Measurements'

Using fluorescent dyes with a modern fluorometer provides an effective tracer system, with the advantages of rapid, accurate, and easy use in the field. Techmation is offering a revised, expanded version of the Turner Designs monograph, *Flow Measurements*, a practical field guide for on-site measuring. An earlier edition of the booklet was described by the EPA as a 'particularly valuable reference'.

Time-of-travel studies, channelling detection, weir and flume calibration, and sewer system infiltration can be done in the field with a Turner Model 10 fluoroemeter and tracer dyes. Theory, equipment, and potential problems are discussed in detail. Basic equations are presented and explained, and reviews of the latest references on the subject are included.

*Free copies from Techmation Ltd, 58 Edgware Way, Edgware, Middlesex HA8 8JP, UK. Tel.: 01 958 3111.*

Circle No. 27 on Reader Enquiry Card

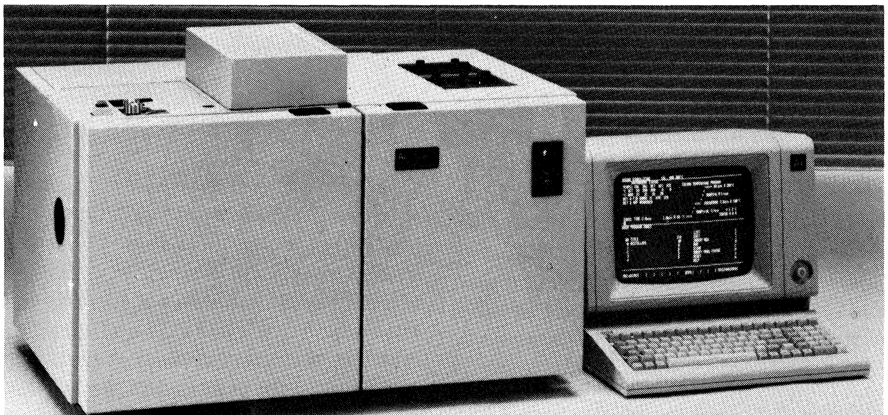

*Model 92—a British designed and built high-performance gas chromatograph from Analytical Instruments Ltd.*

### Bertran introduce high-voltage electronic load

Bertran have announced a high-voltage test instrument for laboratory or inspection use, which provides a convenient way of measuring load regulation, ripple and dynamic response of power-supplies delivering up to 25 kV at 3 mA. The remote programming and monitoring features allow for integration of the new HVL-25 into automatic test equipment systems. The instrument can be operated in several current modes including d.c. operation at either of two current levels, square wave switching between the two current levels at line frequency, or switching between the two current levels at an externally gated frequency.

Current is controlled using two high-voltage vacuum tubes; a dynamic load-balancing circuit eliminates any requirement for expensive selected matched pairs and assures reliable performance.

In addition to the front-panel average current meter a remote average current monitor output is provided. Remote applied voltage, ripple and transient response monitoring (a.c. coupled 330 pf and 10 meg viewing circuit) is also provided.

The instrument is 12 in H × 8 in W × 9 in D and requires 115/230 V, 50/60 Hz (switch selectable) power.

The HVL-25 electronic high voltage load is available off-the-shelf.

*Full information from Bertan at 3 Aerial Way, Syosset, New York 11791, USA. Tel.: 516 433 3110.*

**Circle No. 28 on Reader Enquiry Card**

### Microbiological growth analyser

A number of trade shows are being used to demonstrate System 11 z—a microbiological growth analyser. The analyser provides an automated, standardized method for detecting the presence of micro-organisms by monitoring the change in conductivity caused by their growth in a suitable medium. Data is collected, stored and processed and displayed on a VDU, printer or chart recorder. Software is available to enable blood, urine and other biological fluids to be analysed easily and quickly. Detection of growth is generally faster than by conventional means. The system uses conventional media and autoclavable sample cells in 2, 10 and 100 ml capacities. The power supply cannot be interrupted.

*Details from Malthus Instruments Ltd, William Clowes Street, Burslem, Stoke-on-Trent ST6 3AT, UK. Tel.: 0782 817628.*

**Circle No. 29 on Reader Enquiry Card**

### Oxide etcher

A single-wafer, cassette-to-cassette plasma-processing system for oxide etching, the Plasma Inline 803, has been announced by the Tegal Corporation a subsidiary of Motorola Inc. The 803 features two programmed channels for independent control of all process variables to provide the highest levels of etch repeatability. The advantage of a single-wafer oxide system lies in its ability to handle thickness variations from wafer to wafer that occur in batch processes (for example oxide growth or silicon nitride deposition).

The Inline 803 etches anisotropically or produces sloped profiles for very large scale integration (VLSI). Throughput rates can exceed 60 wafers/h, depending on film type and thickness. The process is compatible with most photoresists, and selectivity to photoresists is typically 4–5 : 1. The 803's reactor and its planar electrode produce a very concentrated plasma close to the surface of the wafer. This ensures optimum etch rates and product uniformity. The reactor electrode is removable for easy cleaning.

The unit has solid state 1000 W RF power supply, a third channel for automatic push-button cleaning, and a high-speed microprocessor-controlled wafer track. Membrane switches actuate the primary controls, and all read-outs are digital. There is also an automatic optical endpoint detection system with programmed overetch for each wafer that detects less than 2% exposed oxide. The system can accommodate all three standard wafer sizes: 3 in, 100 mm, and 125 mm. Other key components include an advanced engineered matching network, automatic vacuum throttling control, and mass-flow controllers for four process gas channels.

*Further information from (UK) TEGAL, 7 Adam Square, Brucefield, Livingston, UK; (USA) TEGAL Corporation, 11 Digital Drive, Novato, California 94947, USA.*

**Circle No. 30 on Reader Enquiry Card**

### ICP analysis

Users of Philips emission analysis systems employing the Boumans inductively coupled plasma source can now make savings in the cost of analysis. Philips are offering a simple, fast method of inserting merely a new torch tip instead of replacing the complete torch. Very often, accidents in the ignition of plasma, increasing the power during running or changing gas flows while operating, will result in the melting of the top of the torch. Furthermore, the deposition of deleterious substances or the erosion of the top of the torch can alter the electrical characteristics of the plasma. Inserting a new tip involves a down time of 10 min and costs a fraction of the price of a new torch.

*Contact Pye Unicam Ltd at York Street, Cambridge CB1 2PX, UK. Tel.: 0223 358866 for more information.*

**Circle No. 31 on Reader Enquiry Card**

### High-speed centrifuges

An illustrated brochure is available describing Beckman's J2-21 series high-speed centrifuges (they are all UK made). The latest model J2-21 M is featured in the catalogue: this is microprocessor-controlled and has the advantage of the Beckman 'ultra-smooth' induction drive, providing high torque for faster acceleration and a more rapid throughput than has previously been available on the J2-21.

The brochure also includes details of the J2-21 itself, and there is a special section on the wide range of standard and special rotors offered by Beckman.

*Copies from Beckman-RIIC Ltd, Progress Road, Sands Industrial Estate, High Wycombe, Buckinghamshire, UK.*

**Circle No. 32 on Reader Enquiry Card**

### Mobile centrifuges

Intended for the chemical, pharmaceutical and nuclear industries, as well as all types of scientific laboratories, a new range of trolley-mounted vertical axis centrifuges from Rousselet can be supplied with a plain bowl, perforated basket, filtration bag and basket or sets of test-tube buckets. They are mobile and have multi-use potential as driers, decanters or separators. Special bowls are available for high-pressure, vacuum or sterile processes. The hydraulic pack and controls mounted on the trolley with the centrifuge allow speeds to be varied from 0 to 4000 rpm. The bowl or basket and the outer shell of the centrifuge can be made of stainless-steel or hastelloy and plated both inside and outside in lead, ebonite, rilsan, teflon, halar or kynar. All specifications are available in two bowl diameters, 40 and 50 cm, 4 and 5·5 kW electric hydraulic drive. Weights are typically 650 and 850 kg.

*Enquiries in UK to Rousselet et Cie S.A., 20 Coniston Road, Linslade, Leighton Buzzard, Bedfordshire LU7 7QY. Tel.: 0525 381569.*

**Circle No. 33 on Reader Enquiry Card**

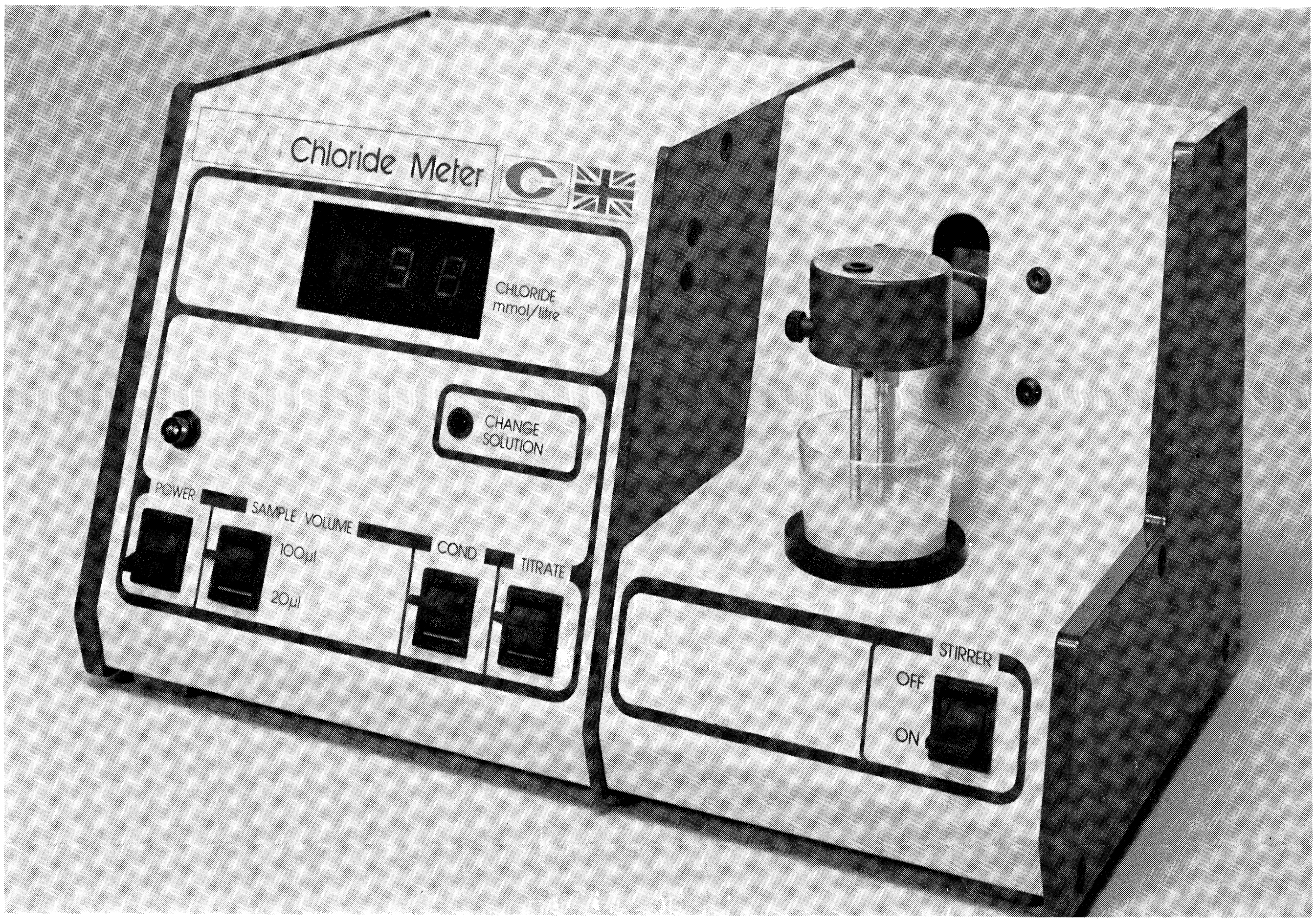

*ChemLab Model CCM.1 chloride meter. This all-British machine provides a fast, accurate digital read-out of the chloride concentration in biological samples such as serum, plasma, sweat, CSF and urine. It is a small, compact instrument with simple controls. Up to 20 samples can be measured in one aliquot of acid buffer solution; an integral warning light indicates when the chloride concentration of the solution has reached the 2000 mmol/l level and needs replacing. The CCM.I is supplied with electrodes, detectors, pipettes, stirring bars and an initial supply of standards, reagents, electrode polish and beakers (UK price is £895). Details from ChemLab Instruments Ltd, Hornminster House, 129 Upminster Road, Hornchurch, Essex RM11 3XJ, UK.*

Circle No. 34 on Reader Enquiry Card

### Pipette tip

The range of precision pipette tips, available from Elkay Laboratory Products (UK) Ltd, has been extended to include cadmium-free tips. Specifically designed for use with Pipetman Liquid transfer pipettors, the tips fit models P-20, P-100, P-200 and F up to 200 µl.

To correspond to the pipettor manufacturers' specification, the tips, which have a capacity of 1–200 µl, are coloured yellow. They are packaged in the Elkay 'Conve-Rack' pack, consisting of five autoclavable trays holding 200 tips, each with integral tray and pack covers. Through the use of these trays, the tip can be attached directly to the pipettor without manual handling: this ensures that the tip is chemically clean before drawing the sample.

*Details from Elkay Laboratory Products (UK) Ltd, Unit 2, Crockford Lane, Basingstoke RG24 0NA, Hampshire, UK. Tel.: 0256 850497.*

Circle No. 35 on Reader Enquiry Card

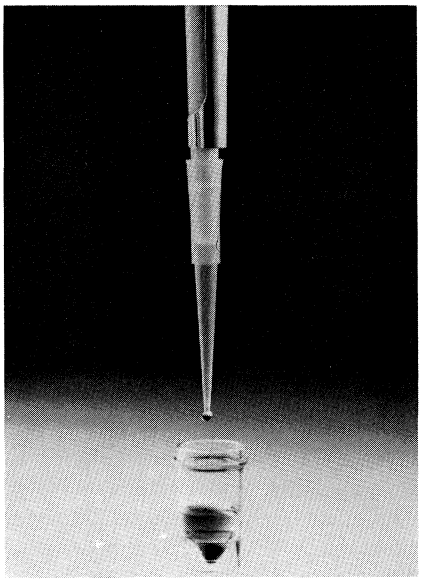

*The new precision pipette tip from Elkay; they are available in individually wrapped sterile packs and in bulk bags of 1000, as well as in the Company's 'Conve-Rack' pack. (Elkay, Basingstoke, UK.)*

### Apple analyser

LabLogic have a multichannel analyser which has been developed for applications such as particle-size distribution analysis. Many models of, for example, blood platelet counters, count in a fixed window of size but research workers may well be interested in looking at the whole distribution of size range. Similar needs exist in nuclear spectroscopy and for users of cytofluorographs.

The unit is based on an Apple microcomputer, there are no modifications to the micro bar the addition of an acquisition board and the use of specialized programs. The programs are written in compiled PASCAL and respond quickly. The multichannel analyser may be purchased to add to an existing Apple or the system may be bought complete.

*For further details contact LabLogic, 72 Eldon Street, Wellington Street Industrial Estate, Sheffield S1 4GT, UK. Tel.: 0742 755085.*

Circle No. 36 on Reader Enquiry Card

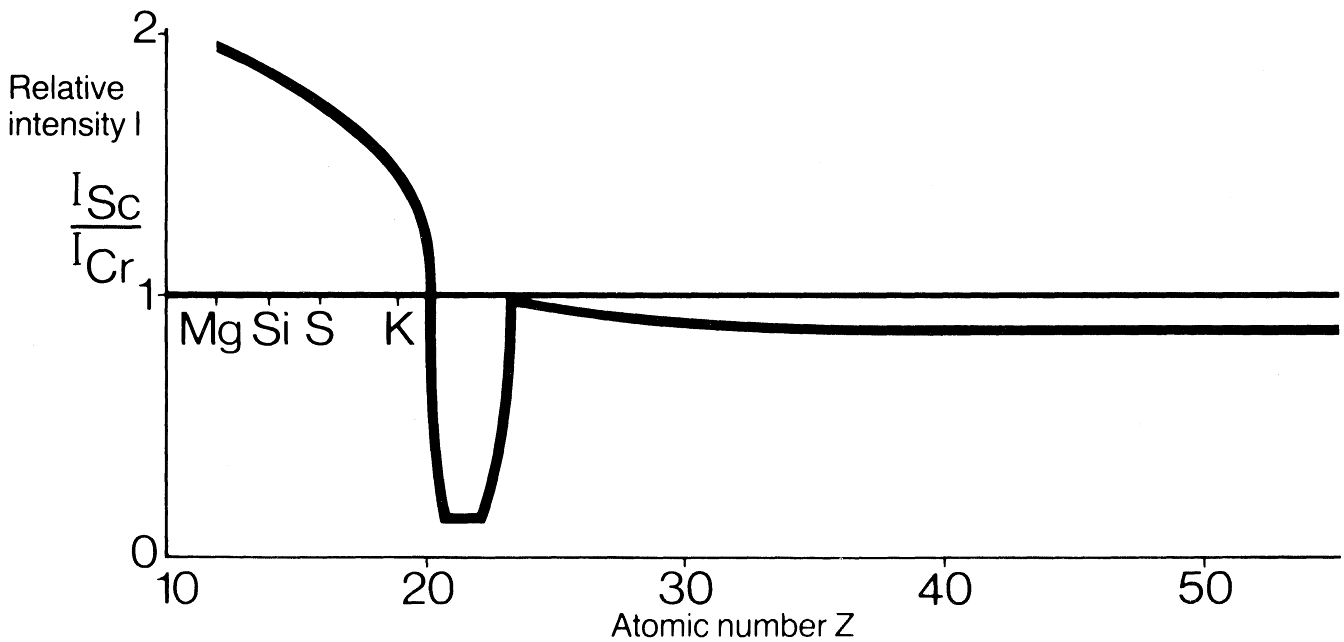

*Graph showing the excitation capability of Philips's scandium-anode X-ray tube, compared with that of a chromium tube. The anode greatly enhances excitation of light elements up to calcium (atomic number 20), while at the same time maintaining excellent results from heavy element analyses. So the new PW2180 model emerges as an attractive alternative to the chromium tube for general-purpose use. Apart from the anode material it is identical in construction with all other Philips side-window tubes and is freely interchangeable in any of the company's sequential spectrometers. Information from Pye-Unicam Ltd, York Street, Cambridge CB1 2PX, UK.*

Circle No. 37 on Reader Enquiry Card

### U-NET for BBC micro

The U-NET Micronetwork system is now being shipped for the BBC micro. U-NET is U-Microcomputers' product for low-cost markets and was previously available for the Apple II only.

U-NET is a shared resources network allowing up to 32 satellite micros to share up to six disk drives and up to two printers. A full printer spooling system is provided on both printers and the whole philosophy is to provide resources to the user as if he were the only user.

*Literature is available from U-Microcomputers Ltd, Winstanley Industrial Estate, Long Lane, Warrington, Cheshire WA2 8PR, UK. Tel.: 0925 54117.*

Circle No. 38 on Reader Enquiry Card

### DNA synthesizer

Using licensed phosphoramidite chemistry with high reactivity, Beckman's System 1 DNA synthesizer has short cycle times and gives a high-quality end-product. The benchtop System 1 offers a choice of complete programmability or step-by-step manual operation. When fully automatic the integral memory of the synthesizer and easy-to-use mini cassettes execute the program, and can synthesize 15 bases or more automatically—each base addition occurring within 30 min.

As a combination of automatic and manual operation, the System 1 can program portions of the synthesis to run automatically, with any number of steps to be controlled manually. A run can be interrupted, with the succeeding step or steps altered before resuming the program.

A wide range of instruments for use in recombinant DNA methodologies is available from Beckman, including an HPLC system for the analysis and purification of synthesized oligonucleotides. System 1 includes the DNA synthesizer with a Beckman HPLC purification system—plus full support in terms of a large selection of reagents and solvents for use in DNA synthesis.

*Further information from Beckman-RIIC Ltd, Progress Road, Sands Industrial Estate, High Wycombe, Buckinghamshire, UK. Tel.: 0494 41181.*

Circle No. 39 on Reader Enquiry Card

### Electronic balance

Launched at the Weightec Exhibition, Sartorius's Ultra Micro Balance (the 4504 MP8) offers a weighing range of 120 mg to a readability of 0·1 mg. Dial-in weights take the total capacity up to 4·02 g. Features include: remote motorized pan extractor and zero; extra wide weighing range, full electronic tare, automatic calibration, standard RS232 data output, self-checking diagnostic sequencing, and 'operator-set' operating parameters. These are possible because the balance includes the MP8 microprocessor developed by Sartorius and which contains all the primary and secondary functions. A major advance which the MP8 provides is an almost unmatchable stability of read-out.

*Full details from Sartorius Instruments Ltd, 18 Avenue Road, Belmont, Surrey SM2 6JD, UK. Tel.: 01 642 8691.*

Circle No. 40 on Reader Enquiry Card

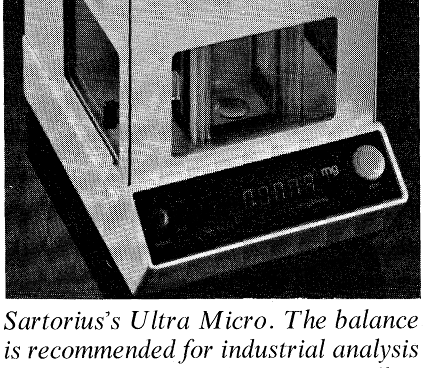

*Sartorius's Ultra Micro. The balance is recommended for industrial analysis in research and development as well as for monitoring working conditions for airborne pollutants.*

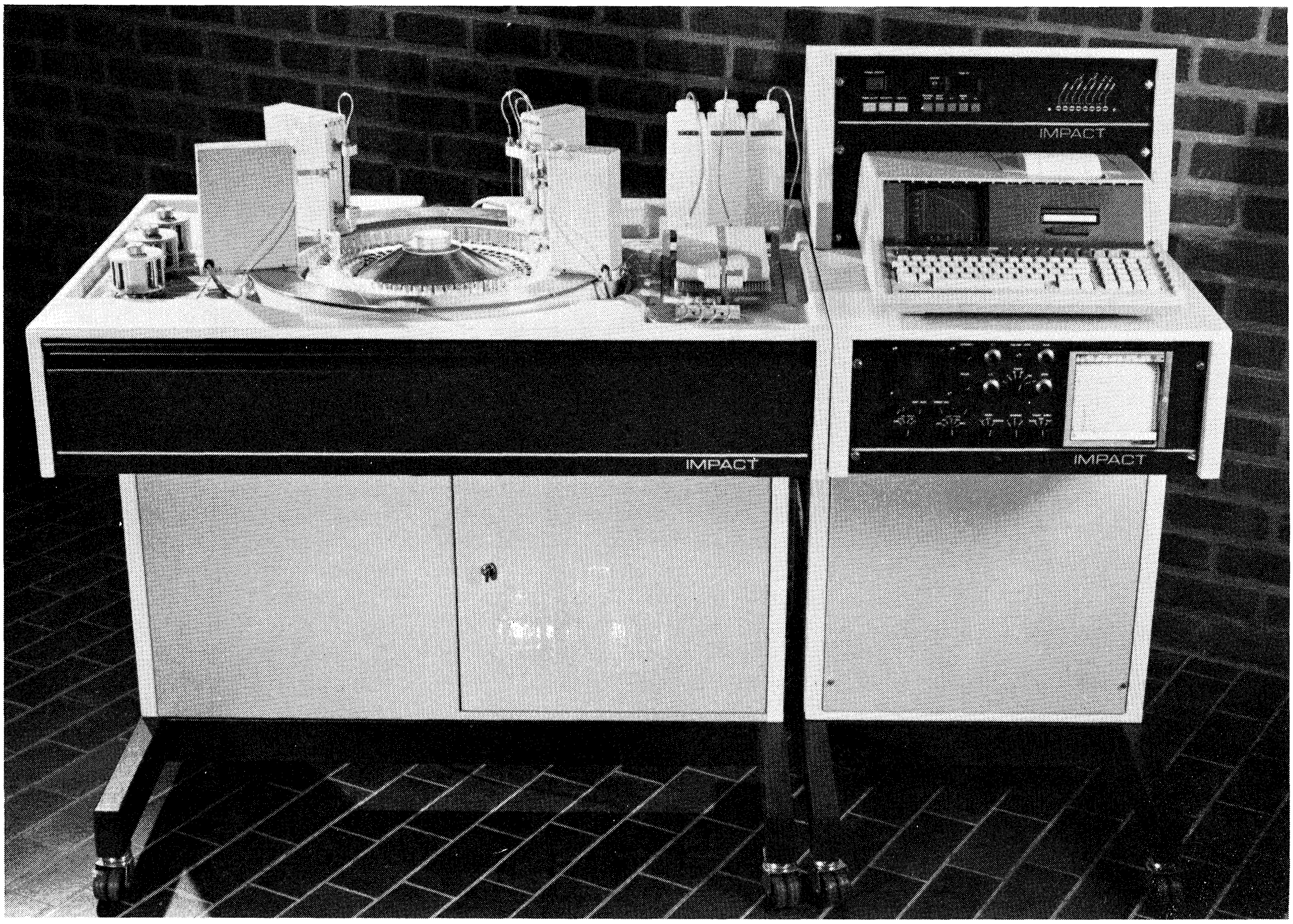

*The first totally automated non-isotopic immunoassay system with a sensitivity and range equal to that of radioimmunoassay. Some 85% of all immunoassays today are performed using radioisotopes. Safety regulations now being drafted in Europe and USA which could price radioimmunoassay out of the market in spite of its enormous diagnostic value. The IMPACT system, with the same capability as RIA, does not use radioisotopes and, as the reagents are totally harmless, disposal problems do not exist, separate laboratories are unnecessary and reagent loss from fast decaying isotopes are eliminated. Further, since the method is totally automated and runs at 60 samples/h, the clinician can obtain immunoassay results together with those for clinical chemistry and haematology, so that correct patient treatment can begin earlier. Technician time is significantly reduced and, depending on the country, can save the laboratory between 25% and 35% on immunoassay. Given some 60 samples/day, a laboratory can pay off the cost of an IMPACT system in less than two years. (ACADE S.A., Brussels.)*

### Automatic non-isotopic immunoassay system

ACADE S.A. was opened during 1983 by the Belgian Minister for Budget, Scientific Policy and Planning. It will be the only company in Belgium to manufacture instruments for *in vitro* diagnostic testing. Their first product is the IMPACT which uses PACIA technology and reagents to fully automate non-isotopic immunoassays. (PACIA stands for Particle Counting Immunoassay.) IMPACT's reagents, because they are non-isotopic, are safe and stable for at least a year. All assays are completed within 1 h. Dr H. W. Holy, the company's Chief Executive Officer, writes that the IMPACT is an important advance in design and technology and that it is fully tested and developed. An ample stock of kits for T4, TBG, ferritin, alphafetoprotein and human placental lactogen is ready for sale. CRP, human growth hormone, IgE, specific IgE antibodies, T3, TSH will follow shortly. Tests such as HCG, circulating immune complexes, streptococcus B, pneumococcus, hemophilus influenza and anti-herpes antibodies can be demonstrated and will become progressively available.

*ACADE are seeking applications from companies of international standing to sell, exclusively, world-wide. Their address is Passage de la Vècquée 17-bte 4100, 1200 Brussels, Belgium.*

**Circle No. 41 on Reader Enquiry Card**

### The CAT

After 45 years' titrator engineering experience, Fisher Scientific have introduced CAT—the Computer-Aided Titrimeter. The aim was to make a system combining a powerful analytical capability with ease of use. Method set-up routines for the system's eight operating modes are menu-driven; the user simply responds to messages on a 20-character display. CAT performs titrations to fixed endpoint targets, titrations with derivative detection of equivalence points, computational methods such as equilibrium and incremental titrations, as well as direct potentiometry in pH, millivolts, or activity with ion-selective electrodes. Titration rate control is automatic, as are electrode standardization and electrode efficiency computation. Test results, entry verification, prompting and status messages appear on vacuum-fluorescent displays.

The system's 'Help' feature uses an accessory printer/plotter to provide a read-out of additional pinpointed instructions whenever required during the set-up routine.

Customized methods can be entered into instrument memory for use when needed; the system also stores stacked sample weights (entered directly from a balance or keyed-in). Stored data can be easily edited. The instrument performs automatic computations for equivalence detection and calculation of activity or concentration in desired units. With the printer/plotter, the system produces printed results, with or without titration curves and thorough documentation of method parameters.

Optional modules can be used to adapt CAT for automated analysis of multiple samples, including programmed pre-treatment.

*For complete information write to Fisher Scientific for a free copy of Bulletin 389F. (Fisher are based at 711 Forbes Avenue, Pittsburgh, Pennsylvania 15219, USA.)*

**Circle No. 42 on Reader Enquiry Card**

### Solid state indicator

A fully solid state indicator, the A 2000, has been added to the Hartmann & Braun range of indicating instruments. The unit has a 100-segment fluorescent display in addition to a three-digit indication of actual value. The A 2000 can be reprogrammed to give a point type or bargraph display; it includes four adjustable programmable alarms which can be remotely set.

Input options are extensive: both voltage and current signals are accepted and the alarm output can be either via switching transistors or potential free relay contacts.

Prices start at around £150·00.

*Hartmann & Braun Ltd are based at Moulton Park, Northampton NN3 1TF, UK. Tel.: 0604 46311.*

**Circle No. 43 on Reader Enquiry Card**

### Applications for Apple and BBC micro

The first series of products from a Norwich-based specialist microcomputer company have been designed for operation with BBC and Apple micros. By using a microcomputer-controlled instrumentation system harnessing the computer's intelligence and standard system components, the performance of traditional stand-alone units is vastly improved. At the same time, instrumentation quality is not compromised, nor do users have to incur the high cost of a duplicated computer system. The first five Oasis products are intended to meet the needs of scientific, educational and industrial research users:

*The Oasis MADC12*—a high-performance data-acquisition module.

*The Oasis SPP 1000*—a microcomputer controlled EPROM programmer with new device personality editor and hardware protection for programmer and EPROM for the Apple and BBC micro.

*The Oasis CADC12*—a high-performance, fully programmable, precision data-acquisition system for the Apple.

*The Oasis FTS card*—a firm time store for the Apple Computer Application designer. This single card gives five-year battery support for 16k bytes of fast access paged RAM and a flexible real time clock. Pages EPROM capacity to 16 k bytes is provided for the designer's firmware.

*The Oasis Diary Card*—a completely self-contained appointment scheduling system for the Apple. Full software, clock facilities and data-base memory are incorporated on a single card. The Oasis Diary Card utilizes memory and clock-support capabilities for the Oasis CFTS16 card.

*Further information from Oasis Electronics Ltd, University Village, Norwich, Norfolk NR4 7TJ, UK. Tel.: 0603 503275.*

**Circle No. 44 on Reader Enquiry Card**

### Sensitive, simple to use spectrofluorometer

A high-energy xenon lamp is used as the light source for Kontron's SFM 25 spectrofluorometer; it also uses an efficient optical system with blazed gratings for maximum energy throughput, and a high-performance photomultiplier. Reproducibility is ensured by reference beam compensation for variations in source output, digital signal processing and continuous dark current compensation.

Nine sets of complete method parameters can be stored in the memory; once a set has been selected the start button is pressed and the results can be read.

Four wavelength operating modes are available—fixed wavelength, excitation scan, emission scan and synchroscan, and these allow the SFM 25 to be used for a wide range of fluorescent analytical techniques. With fixed wavelength operation, quantitative measurements of concentration can be swiftly performed with automatic subtraction of solvent blank values. In excitation and emission scan modes, the SFM 25 can be used to determine optimum wavelengths for quantitative analysis and characterize unknown substances. Synchroscan greatly assists the identification of organic compounds and the spectrum produced can serve as a 'fingerprint' for complex mixtures such as raw oil. Two basic versions of the spectrofluorometer are available: one with a four-cell holder and one with a flow cell for HPLC. The HPLC flow cell holder has high sensitivity, low response volume and ideal flushing characteristics; mounted on its own base, it is easily removed for cleaning. Thermostatted cell holders are also offered for biological applications of fluorescence where they can improve both sensitivity and reproducibility.

Standard to all of the spectrofluorometers is an RS232C interface. This is bi-directional and allows for the print-out of parameters and results, as well as full outside control. Baud rate, parity and format are all set up via the SFM 25's keyboard.

*Further details from Kontron Analytical, PO Box 88, St. Albans AL1 5JG, Hertfordshire, UK. Tel.: 0727 66222.*

**Circle No. 45 on Reader Enquiry Card**

### Balances

Salter Industrial Measurement Ltd, a subsidiary of Stavely Industries PLC, offer a number of electronic precision balances; the range has two basic categories—balances accurate up to one part in 5000 and high-precision balances accurate up to one part in 20 000. The line contains (model No., balance, increments):

| | | |
|---|---|---|
| EB 500: | 500 g; | 0·1 g |
| EB 5000: | 5000 g; | 1 g |
| PB 220: | 20 g/200 g; | 1 mg/10 mg (dual range scale) |
| PB 2200: | 200 g/2000 g; | 10 mg/100 mg (dual range scale) |
| PB 200: | 200 g; | 1 mg |
| PB 2000: | 2000 g; | 10 mg |
| PBC 2000: | 2000 g; | 10 mg (counting scale). |

They are all mains powered and a glass wind-break is available for use with 20 g and 200 g capacity machines. Guaranteed for 12 months, the balances range in price from £1000 to £5000 plus VAT.

*Contact Salter Industrial Measurement Ltd, at George Street, West Bromwich, West Midlands B70 6AD, UK, for more information. Tel.: 021 454 9211.*

**Circle No. 46 on Reader Enquiry Card**

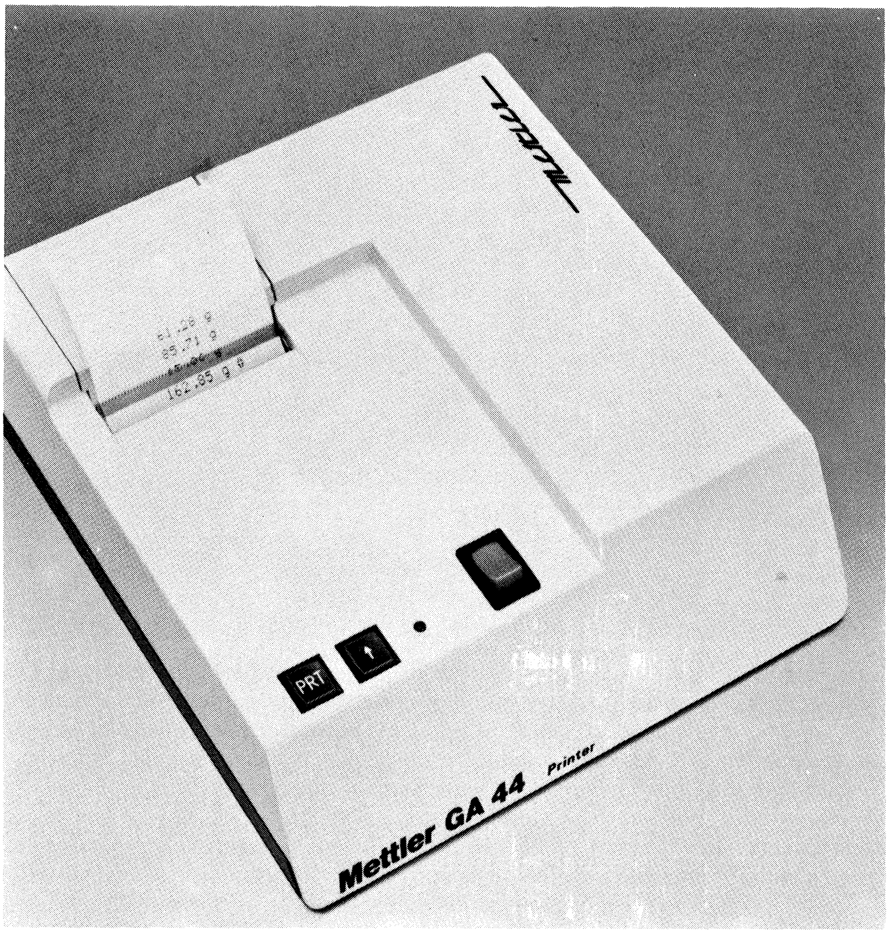

*The GA44 thermal printer—a significant improvement over manual recording of weighing results because it saves time and avoids transcription errors. It can be connected to all Mettler electronic balances with serial data output. Measuring values are printed-out with the correct sign in front and the proper unit designation. Appropriate parameters in each line assure automatic identification of all values. The capacity of the GA44 is 20 characters/line; the transfer rate is adjustable in four steps from 110 to 2400 baud. Details from Mettler Instrumente AG, CH 8606 Greifensee, Switzerland.*

**Circle No. 47 on Reader Enquiry Card**

### Cryostat

A general-purpose cryostat featuring a retracting microtome and a wide self-defrost chamber is available from Slee's Laboratory Equipment Division (it's called the HR cryostat).

The R2 rotary microtome is extremely rigid, completely rust-proof and designed to be self-compensating to keep it independent of any temperature variations. It has stainless-steel, frictionless bearings and enables sections to be precisely cut from 1 to 25 $\mu$m. The self-aligning, easy to adjust guide plate ensures flat, uniform and crease-free sections.

An important feature of the HR cryostat is balanced manual drive with a safety lock on the top of the stroke for the microtome.

The chamber, with its large clear-view window and access port, enables relaxed user operation whilst the circuitry and component layout of the cooling system ensure reliability.

A hermetically sealed compressor gives a range of 0° to −35°C, dependent upon the refrigerant gas employed and ambient temperature.

With a voltage requirement of 240/110 V and 50/60 Hz operation, the HR cryostat is recommended for routine histology, research or teaching and for cutting all types of histological, cytological and technological materials.

Accessories include a bench freezer and tools for rapidly freezing specimens, microtome knives, and a range of specimen holders.

*Details from the Slee Group's Laboratory Equipment Division, Lanier Works, Hither Green Lane, London SE13 6QD, UK.*

**Circle No. 48 on Reader Enquiry Card**

### Free electrodes

In an attempt to expand the number of methods for ion-selective electrodes into new areas Orion are running a competition for their customers. So every customer supplying a new method (that they have developed themselves) will, as long as the method is accepted, get a free electrode (the model 97-08 is excluded). Acceptance will be based on two things:

(1) The method must be new.
(2) The method must be applicable to other users.

*Methods to Mrs S. M. Chick, MSE Instruments, Manor Royal, Crawley, Sussex RH10 2QQ, UK.*

### *New methods*

Orion's Technical Services Group have announced six new methods (available from MSE in the form of 'technical service notes'):

Nitrate in potable and ground waters.
pH of water samples (these notes discuss the typical problems encountered and what to do to solve them).
Soil pH (three methods are given, along with recommendations for electrodes).
Calcium in feed-stuffs (an important measurement for determining a key nutrient).
Acidity and alkalinity determinations (an ideal application for the Ross electrode).
Titrations for silver in photographic solutions.

### *Ammonia*

Orion have released a new ammonia electrode, which carries a two-year warranty. The 95-12 is a precision-manufactured device offering greater reliability and smaller in size than the old design. Smaller samples can be measured, even in test-tubes. The simplified assembly eliminates the need for 'O' rings and spacers, thus reducing the assembly problems commonly associated with gas-sensing electrodes.

**Circle No. 49 on Reader Enquiry Card**

### Hydrocarbons and GC

Towards the end of 1983, the PolyScience Corporation of Niles, USA published Seaton T. Preston, Jr.'s and Ronald E. Pankratz's *Guide to the Analysis of Hydrocarbons by Gas Chromatography*. The 361-page book is an analyst's introduction and reference to hydrocarbon analysis by gas chromatography; a summary is presented of qualitative and quantitative analysis techniques for hydrocarbons, and of factors influencing the selection of liquid phases and column support materials. The important physical properties and chemical structures for many commonly encountered hydrocarbons are included for reference. The central section of the book presents 210 pages of tabular retention data compiled from the literature; the liquid phases for all studies included are indexed and all data sources are fully referenced. The bibliography section lists 1061 references published during 1981 relevant to hydrocarbon analysis.

*The book is priced at $45·00 (soft cover); orders (ISBN 0-913106-21-6) to PolyScience Corporation, 7800 Merrimac Avenue, PO Box 48312, Niles, Illinois 60648, USA.*

Circle No. 50 on Reader Enquiry Card

### Sonic concentration analyser

Model 6380 includes many of the features of Mapco's model 6180 whilst considerably reducing the cost. The analyser will control the concentration of acids, salts, bases, alcohols, polymer reactions and other binary organic and aqueous solutions to within a hundredth of a percent. The 6380 is provided with a standard temperature-compensated 4-20 mA isolated output and is packaged in a NEMA 4 × fibre glass enclosure. Transducer assemblies capable of withstanding temperatures up to 260°C and pressures up to 3000 psi are available in stainless steel, Carpenter 20, Hastelloy B, Hastelloy C and Titanium.

*More information from Mapco Controls Company, 11391 East Tecumseh, PO Box 21418, Tulsa, Oklahoma 74121, USA. Tel.: 918 438 1010.*

Circle No. 51 on Reader Enquiry Card

### Monitoring photosynthesis

Precise determination of changes in carbon dioxide and water vapour levels in plant chambers is possible with the BINOS infra-red gas analyser. The measurement range is ±25 ppm $CO_2$ at a nominal reference point value of 330 ppm $CO_2$. It also measures absolute $CO_2$ (typically 0 to 600 ppm) and absolute and differential $H_2O$ (0 to 1% as an absolute range and 0 to 5000 ppm as a differential range).

*Further information from Tekmar Company, PO Box 371856, Cincinnati, Ohio 45222, USA.*

Circle No. 52 on Reader Enquiry Card

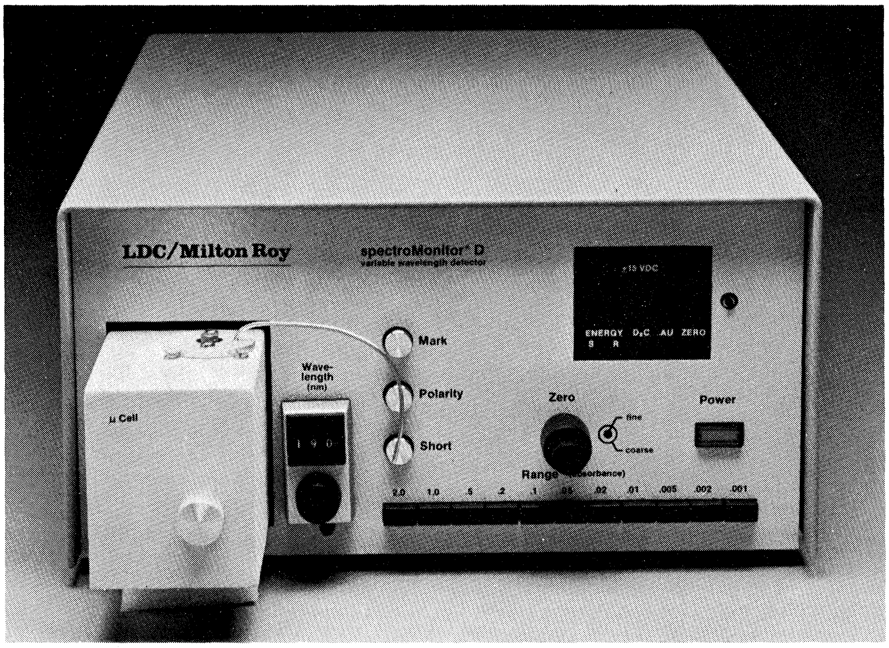

*The microCell fluid cell by LDC/Milton Roy is designed for microbore HPLC applications. It features a unique cross-flow optical design, minimizing thermal and flow noise without a heat exchanger. A tubular (1 mm bore) fused-silica cell permits direct coupling to 1 mm bore columns for maximum chromatographic efficiency. Special fibre-optics and a high performance mirror direct light through the cell on to an integral pre-amplifier circuit board. The microCell has a low noise specification: less than $4 \times 10^{-5}$ Au (at 220 nm). It can be retrofitted to spectroMonitor variable wavelength detectors; alternatively, the spectroMonitor D can be purchased with a factory-installed microCell. Details from LDC, Milton Roy House, High Street, Stone, Staffordshire ST15 8AR, UK. Tel.: 0785 813542.*

Circle No. 53 on Reader Enquiry Card

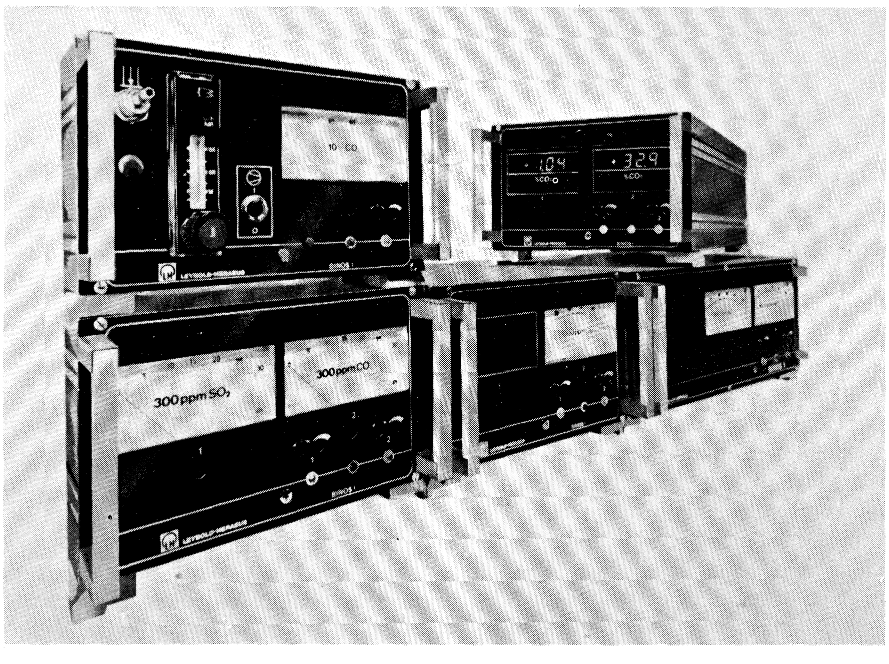

*Four standard models of the BINOS infra-red gas analyser are available to the photosynthesis researcher and all four have appropriate infra-red optics to maximize accuracy and sensitivity. Each model is portable and works on 12 V d.c. or 120 V a.c.; heated optics are not required. The models feature analogue or digital display of the measured component and provide a 0 to 1 V recorder output signal over the measured range. (Tekmar, USA.)*

